# AGE AT CARDIO-METABOLIC DISEASE ONSET IN A COHORT OF MIDLIFE WOMEN: SYSTEMATIC EXCLUSION MISESTIMATES THE MAGNITUDE OF RACIAL DISPARITIES

**DOI:** 10.1093/geroni/igac059.326

**Published:** 2022-12-20

**Authors:** Alexis Reeves, Michael Elliott, Tene Lewis, Carrie Karvonen-Gutierriez, William Herman, Sioban Harlow

**Affiliations:** Stanford University, School of Medicine, Berkeley, California, United States; University of Michigan, Ann Arbor, Michigan, United States; Emory University, Atlanta, Georgia, United States; University of Michigan, Ann Arbor, Michigan, United States; University of Michigan, Ann Arbor, Michigan, United States; University of Michigan, Ann Arbor, Michigan, United States

## Abstract

Cohort studies of aging recruit participants at an age before most of the population experiences the study outcome, to document its natural history. The age of study commencement is usually based on “normative” aging among Whites. However, “weathering” can cause accelerated health declines in minoritized populations compared to Whites due to cumulative experience of multiple forms of marginalization. Thus, considering if weathering among minoritized individuals could affect selection into cohort studies is necessary to effectively estimate and understand racial/ethnic disparities in aging and disease. Using the Study of Women’s Health Across the Nation (SWAN), a multi-ethnic longitudinal cohort, and its cross-sectional screening survey, we examine the effects of selection on the racial/ethnic differences in the age of onset of 4 cardio-metabolic outcomes (hypertension, isolated systolic hypertension, insulin resistance and diabetes). Selection at study commencement (left truncation and left censoring) had greater effects on outcomes with earlier age at onset (hypertension) and right censoring had greater effects on outcomes with later onsets (metabolic). Full adjustment led to an average 20-year decrease in predicted median age of onset for all groups across the 4 outcomes and tended to decrease the predicted disparity in age at onset. However, significantly earlier onset of each outcome for Black and Hispanic women compared to Whites remained. Not considering the full extent of selection bias in cohort studies can misinform our understanding of aging and disease, especially for minoritized populations who have higher prevalence of these leading causes of morbidity and mortality earlier in life.

